# Prenatal Drug Exposure in Children With a History of Neuropsychiatric Care: A Nested Case-Control Study

**DOI:** 10.3389/fpsyt.2022.795890

**Published:** 2022-03-22

**Authors:** Justine Benevent, Caroline Hurault-Delarue, Mélanie Araujo, Alexis Revet, Agnès Sommet, Isabelle Lacroix, Christine Damase-Michel

**Affiliations:** ^1^Department of Medical and Clinical Pharmacology, Toulouse University Hospital (CHU de Toulouse), CERPOP - SPHERE Team, Inserm, Toulouse, France; ^2^Department of Child and Adolescent Psychiatry, Toulouse University Hospital (CHU de Toulouse), Toulouse, France

**Keywords:** POMME cohort, neuropsychiatric disorders, child, medication, prenatal exposure, delayed effects, pharmacoepidemiology

## Abstract

**Background and Objectives:**

Neuropsychiatric disorders in childhood after prenatal drug exposure raises concerns. Most of the published studies focused on psychotropic medications. This study investigated which prenatal medication exposure was associated with neuropsychiatric disorders in childhood.

**Methods:**

A case-control study, nested in the French POMME cohort, was conducted to compare prenatal medication exposure between children with a history of neuropsychiatric care (ages 0–8 years) and children in a control group. POMME included children born in Haute-Garonne to women covered by the general Health Insurance System, between 2010 and 2011 (*N* = 8,372). Cases were identified through: (1) reimbursement for neuropsychiatric care; (2) psychomotor development abnormalities specified on health certificates; and (3) reimbursement for methylphenidate or neuroleptics. Controls had none of these criteria. Prenatal exposure to each of the major “Anatomical Therapeutic Chemical” classes was compared between the groups. Class(es) for which there was a statistically significant difference (after Bonferroni adjustment, i.e., p < *0.0033*) was(were) compared using logistic regression.

**Results:**

A total of 723 (8.6%) cases and 4,924 (58.8%) controls were identified. This study showed a statistically significant difference in prenatal exposure to nervous system drugs (excluding analgesics) between the groups [ORa: 2.12 (1.55; 2.90)]. Differences (not statistically significant at the 0.0033 threshold) were also observed for the ATC classes: Musculoskeletal, Genito-urinary System and Sex Hormones, Alimentary Tract and Anti-infectives.

**Conclusion:**

Through identification of children with neuropsychiatric disorders and of their prenatal medication exposure, this study provides guidance for the assessment of long-term neuropsychiatric effects after prenatal medication exposure, without focusing on psychotropic medications.

## Introduction

Neuropsychiatric disorders are a diverse group of conditions that include both neurodevelopmental disorders and other psychiatric disorders. Based on the fifth edition of the Diagnostic and Statistical Manual of Mental Disorders (1), neurodevelopmental disorders include autism spectrum disorder (ASD), attention deficit/hyperactivity disorder (ADHD), communication disorders, developmental coordination disorder and learning disabilities. Other psychiatric disorders include anxiety disorders, mood disorders, childhood schizophrenia, etc. Epidemiological data have shown an increase in the prevalence of childhood neuropsychiatric disorders in recent years (2, 3). The origin of neuropsychiatric disorders is multifaceted and certain events that occur during intrauterine life may contribute to the risk of these disorders (4). For example, prenatal exposure to valproic acid has been shown to increase the risk of ASD in children (5). Recently, the involvement of other psychotropic medications in delayed neuropsychiatric effects has been examined and the results are conflicting (6–11). Moreover, nearly all studies on the subject have focused on psychotropic drugs, yet many medications cross the placenta and could be responsible for long-term effects on brain function. For example, studies have highlighted associations between childhood neurodevelopmental disorders and prenatal exposure to glucose-lowering medications, folic acid or immunosuppressants (12–14).

The objective of this study was to investigate associations between prenatal medication exposure, psychotropic or not, and care or treatment related to neurodevelopmental or psychiatric disorders in childhood. To address that, a comparison of prenatal medication exposure in children with a history of neuropsychiatric care and control children was conducted in the POMME cohort.

## Materials and Methods

### Data Source

The French POMME [*PrescriptiOn Médicaments Mères Enfants* (Prescription-Drugs-Mothers-Children)] cohort [described in a previous article (15)] was used to conduct the study. POMME holds anonymized data on children from conception and during their childhood. POMME records data from two sources.

First the French Health Insurance System yearly provides with all data on medicines prescribed and reimbursed to the mother during pregnancy (medicine exposure during intrauterine life) and with data on medicines and medical care prescribed and reimbursed to the children during childhood. In France, the health insurance system is universal and manages all reimbursements of health care for all people affiliated to a health insurance scheme in France, complemented by mutual funds or private insurance companies. The main system is for salaried workers and covers about 80% of the population. Expenses for most of the medications, except those deemed not to contribute much to health, are partially or totally covered by the health insurance system. The French list of refundable medicines is available on the French Health Insurance System website [4]. Expenses for medical visits and medical care are also covered by the health insurance system. The French health care system is based on a mix of public and private practice and the patients have the freedom of choice when consulting physicians (general practitioner or specialists). Therefore, POMME holds information from both public and private health care systems. All expectant mothers declare their pregnancy to the French Health Insurance System, which records the date of the beginning of pregnancy and of childbirth (sent by maternity services for reimbursement of hospital expenses). During the first 6 months of pregnancy, medications are reimbursed at a rate of 35 or 65%. Thereafter, medical care and medications are provided free of charge until the end of pregnancy. After birth, children are generally covered by their mother's health insurance.

Second, the Maternal and child protection service provides data from children's certificates filled out during the compulsory medical examinations at birth, 9 and 24 months of age. The examinations are performed by a general practitioner or a pediatrician, using the standardized questionnaire forming the certificates. Then, the parents have to send certificates under confidential cover to the Mother and Child Protection Centre.

POMME includes children born to women covered by the general health insurance scheme, between July 1, 2010 and June 30, 2011. POMME is updated annually. Currently, available data concern children until 8 years of age.

### Study Design

We conducted a retrospective case-control study nested in the POMME cohort. Prenatal exposure to each of the major ATC classes was compared between cases and controls.

### Population

The initial population was live-born registered in the POMME cohort. We excluded children born with chromosomal abnormality(ies). The case and control groups were then formed by applying inclusion criteria related to (1) medical visits to a neuropsychiatric specialist, (2) reimbursement for neuropsychiatric cares, (3) items of psychomotor development on the health certificates, (4) neuropsychiatric condition specified on the health certificate, (5) reimbursement for methylphenidate and/or neuroleptics and (6) prescription of medications by a neuropsychiatric specialist. Inclusion criteria for cases and controls are presented in Table 1.

**Table 1:** Selection criteria for the case and control populations.

	**Cases**	**Controls**
Medical visits	At least two reimbursements for visits to a neuropsychiatric specialist (neuropsychiatrist, psychiatrist, pediatric neurologist or child psychiatrist)	No reimbursement for visit to a neuropsychiatric specialist (neuropsychiatrist, psychiatrist, pediatric neurologist or child psychiatrist)
Neuropsychiatric cares	At least one reimbursement for: - Hospitalization in a child psychiatry department - Care in a psychoeducational medical center - Screening for cognitive or intellectual deficits, depression, psychopathological aspects of personality or adaptation and psychosocial functioning disorders	No reimbursement for: - Hospitalization in a child psychiatry department - Care in a psychoeducational medical center - Screening for cognitive or intellectual deficits, depression, psychopathological aspects of personality or adaptation and psychosocial functioning disorders
Items of psychomotor development on the heath certificates	At least two negative non-motor items of psychomotor development on the 9th health certificate and/or two negative non-motor items on the 24th health certificate	No negative non-motor item of psychomotor development on the 9th health certificate or on the 24th health certificate
Neuropsychiatric condition specified on the health certificate	At least one neuropsychiatric condition (psychomotor, language or developmental delay/retardation, or communication and interaction disorders) specified on the open field on at least one of the health certificates	No neuropsychiatric condition specified on the open field on at least one of the health certificates
Use of methylphenidate and/or neuroleptics	At least one reimbursement for methylphenidate and/or neuroleptics (ATC class N05A)	No reimbursement for methylphenidate and/or neuroleptics (ATC class N05A)
Prescriber specialty		No reimbursement for medications prescribed by a neuropsychiatric specialist

### Exposures

Prenatal medication exposure was estimated based on outpatient dispensing of prescribed and reimbursed medications to mothers during pregnancy. Prenatal medication exposure was described according to the ATC (Anatomical, Therapeutic, Chemical) classification (16). A child was considered to be prenatally exposed to one of the ATC classes if at least one medication belonging to the class was dispensed to the mother during pregnancy. Prenatal medication exposure was also described according to the trimester of pregnancy [defined as: first trimester: from conception to week 12+6; second trimester: from week 13 to week 25+6; third trimester from week 26 until the end of pregnancy].

### Outcome

The outcome was any history of neuropsychiatric care in children between 0 and 8 years of age, identified through five criteria (Table 1). Children were considered as having a history of neuropsychiatric care if they had (1) at least two reimbursements for visits to a neuropsychiatric specialist (neuropsychiatrist, psychiatrist, pediatric neurologist or child psychiatrist); and/or (2) at least one reimbursements for a neuropsychiatric care (such as hospitalization in a child psychiatry department); and/or (3) at least two negative non-motor items of psychomotor development on the 9th and/or 24th health certificates; and/or (4) at least one neuropsychiatric condition (i.e., psychomotor, language or developmental delay/retardation,) specified on the open field on at least one of the health certificates; and/or (5) at least one reimbursement for methylphenidate and/or neuroleptics (ATC class N05A). Indeed, those psychotropic medications are only used in the indication of neuropsychiatric disorders.

### Statistical Analyses

The analyses were carried out using SAS^®^ version 9.4 software (SAS Institute Inc., Cary, NC, USA).

#### Descriptive Analyses

Characteristics of case and control subjects, such as gender, prematurity (birth before 37 weeks of amenorrhea) and the presence of congenital malformation(s) coded according to the International Classification of Diseases 10th Revision (ICD-10) (17), were described. Characteristics of their parents (maternal age, maternal education, and parents who have no profession) were also presented. Categorical variables were described by the frequency and proportion of each modality and quantitative continuous variables by mean and standard deviation.

#### Comparative Analyses

The first step was a comparison between cases and controls for prenatal exposure to each of the major ATC classes using a chi-square (χ^2^) test or a Fisher test. Exposure to the ATC “Nervous System” class was described in different ways: excluding analgesics, including analgesics and excluding acetaminophen. As we carried out multiple comparisons (13 major ATC classes and 2 subgroups of the “Nervous System” class), we used a Bonferroni correction and the significance threshold of statistical tests (*p* = *0.05*) was divided by 15 to establish whether or not the tests had a significance level (i.e., *p* < *0.0033*) in order to take into account the increase in alpha risk.

In the second step, we focused on the ATC class(es) for which we observed a statistically significant difference of prenatal exposure between cases and controls in the first step. Multivariate logistic regression analyses were performed to evaluate the association between prenatal exposure to this(these) classe(s) and a history of neuropsychiatric care in childhood, to adjust for potential confounding factors. Adjusted model(s) with significant covariates were constructed using backward selection with the likelihood ratio test. The statistical significance level *p* was 0.05.

### Adjustment Variables

Certain factors that might be associated with neuropsychiatric disorders were identified and included in our logistic regression models. For mothers, we considered age, parity, professional status, long-term disease [chronic condition specifically listed by the French healthcare system as a long-term disease (18)], smoking and hospitalizations during pregnancy for high blood pressure, preterm labor or intrauterine growth retardation. Regarding medical data on pregnancies, we considered multiple pregnancies and the number of follow-up ultrasounds during pregnancy. Finally, for children, we considered gender, prematurity, breastfeeding, congenital malformation, abnormal hearing test, anthropometric data at 8 days and 9 and 24 months (weight, height and head circumference) and health data during the first week of life (pathology, antibiotic therapy, intubation, and oxygen therapy).

## Results

Among the 8,372 children included in the POMME cohort, a history of neuropsychiatric care from 0 to 8 years of age was identified in 723 (8.6%) children, who constituted the case population. The control population, i.e., children who had no history of neuropsychiatric care, consisted of 4,924 children (58.8%).

### Description of the Case and Control Subjects

#### Description of the Characteristics of the Case and Control Subjects

Table 2 presents the characteristics and health status of the case and control subjects and the characteristics of their parents. A majority of the case subjects were male (67.5 vs. 48.1% of the controls (*p* < 0.0001)). The proportions of prematurely born children and of children with abnormal hearing test were higher in the case group than in the control group [respectively 6.9 vs. 5.6% (*p* = 0.1238) and 7.4 vs. 4.3% (*p* = 0.0045)], as well as the proportions of parents who “had no profession” [29.8% of the mothers vs. 25.6% (*p* = 0.0011) and 15.1% of the fathers vs. 13.2% (*p* = 0.1689)].

**Table 2:** Characteristics of the case and control subjects.

	**Cases** ***N*** **=** **723**		**Controls** ***N*** **=** **4,924**		***P***
	**Sample size**	***N* (%)**	**Sample size**	***N* (%)**	
Male gender	723	488 (67.5)	4,924	2,369 (48.1)	<0.0001
Prematurity (birth before 37 weeks of amenorrhea)	723	50 (6.9)	4,924	275 (5.6)	0.1238
Breastfed child	680	486 (71.5)	4,580	3,301 (72.1)	0.7144
APGAR at 1 min ≤ 7	588	25 (4.3)	4,162	204 (4.9)	0.7555
APGAR at 5 min ≤ 7	652	7 (1.1)	4,468	45 (1.0)	0.8501
Pathology during the 1st week of life	667	57 (8.5)	4,587	303 (6.6)	0.0120
Oxygen therapy during the first week of life	667	18 (2.7)	4,587	116 (2.5)	0.1975
Abnormal hearing tests between 0 and 2 years of age	353	26 (7.4)	1,919	82 (4.3)	0.0045
Congenital malformations	723	37 (5.1)	4,924	89 (1.8)	<0.0001
Mothers' level of education	342		2,411		
Primary school		6 (1.8)		37 (1.5)	0.7456
High school		50 (14.6)		305 (12.7)	0.2603
High school diploma level		83 (24.3)		536 (22.2)	0.3572
Higher education		203 (59.4)		1,533 (63.6)	0.1029
Mother without profession	701	209 (29.8)	4,695	1,204 (25.6)	0.0011
Father without profession	624	94 (15.1)	4,023	531 (13.2)	0.1689
Smoking during pregnancy	321	50 (15.6)	1,995	283 (14.2)	0.4799
Multiple pregnancies	723	22 (3.0)	4,924	152 (3.1)	0.9459
	***N***	**Mean (error measures)**	***N***	**Mean (error measures)**	
Maternal age	723	30.8 (5.3)	4,924	30.4 (4.9)	0.0428
Maternal parity	592	1.6 (0.8)	4,055	1.7 (0.9)	0.0017
Head circumference at birth (cm)	620	34.3 (1.8)	4,267	34.4 (1.7)	0.1671
Birth weight (g)	660	3,246.1 (544.1)	4,540	3,278.2 (499.8)	0.1299
Birth height (cm)	626	48.9 (2.6)	4,323	49.1 (2.3)	0.0547

The overall rate of congenital malformations was higher in the case population (5.1%) than in the control population (1.8%) (see Supplementary Table 1). Among the case subjects, the most common congenital malformations were those of the urinary system (165.5/10,000 vs. 28.4/10,000 among controls). The rate of nervous system congenital malformations was higher in the case population (41.4/10,000 vs. 4.1/10,000 among controls).

#### Description of the History of Neuropsychiatric Care Among the Case Subjects

Nearly half of the cases (*N* = 335; 46.3%) had a reimbursement for at least one medical visit to a neuropsychiatric specialist. The mean age of the children at the first medical visit was 4.5 years. The majority of medical visits were with psychiatrists (43.3% of the cases). The specialists consulted the earliest were child psychiatrists, at an average age of 4.4 years. The neuropsychiatric care most often reimbursed (*N* = 151 children) was related to cognitive or intellectual deficits, and 107 children attended sessions in psychoeducational medical centers.

Table 3 presents a description of the health certificate items regarding psychomotor development in the case subjects. The most common negative non-motor item at 9 months was “Does not play Peek-a-boo” (15.0% of the cases) and at 24 months “Does not combine two words” (35.4% of the cases).

**Table 3:** Psychomotor development items for case subjects (*N* = 723).

**Psychomotor development items**	***N* (%)**	**Type of item**
**At 9 months**		
Does not play Peek-a-boo (*N* = 586)	88 (15.0)	Non-motor
Does not repeat syllables (*N* = 597)	71 (11.9)	Non-motor
Does not respond to first name (*N* = 596)	34 (5.7)	Non-motor
Does not point (*N* = 586)	217 (37.0)	Motor
Does not move around (*N* = 596)	170 (28.5)	Motor
Does not sit without a prop (*N* = 599)	43 (7.2)	Motor
Does not grasp objects (*N* = 593)	31 (5.2)	Motor
Lack of limb motricity (*N* = 594)	25 (4.2)	Motor
**At 24 months**		
Does not combine 2 words (*N* = 458)	162 (35.4)	Non-motor
Does not name pictures (*N* = 457)	141 (30.9)	Non-motor
Does not understand a simple instruction (*N* = 459)	18 (3.9)	Non-motor
Does not stack objects (*N* = 457)	21 (4.6)	Motor
Lack of symmetrical motricity in the 4 limbs (*N* = 456)	17 (3.7)	Motor
Is not walking (*N* = 461)	10 (2.2)	Motor

Forty children (5.5% of the cases) were exposed to methylphenidate. The neuroleptics to which children in the case group were exposed to were risperidone (*N* = 9 children exposed), cyamemazine (*N* = 6 exposed) and propericiazine (*N* = 1 exposed).

### Comparison of Prenatal Medication Exposure Between Case and Control Subjects

Table 4 presents the comparisons of prenatal exposure to the different ATC classes between cases and controls. Prenatal medication exposure was higher in the case group than in the control group for all the ATC classes. At a significance threshold of 0.33% (after Bonferroni adjustment), the only ATC class for which there was a statistically significant difference was the Nervous System drugs. The difference between the groups was less statistically significant when analgesics were included (*p* = *0.0011* vs. *p*<*0.0001)*. Exposure to four other ATC classes [Alimentary Tract and Metabolism (A), Genitourinary System and Sex Hormones (G), Systemic Anti-infectives (J) and Musculoskeletal System (M)] was higher in the case group than in the control group but these differences were not statistically significant at the 0.0033 threshold.

**Table 4:** ATC (anatomical therapeutic chemical) classes to which the case and control subjects were exposed *in utero*.

	**ATC (anatomical therapeutic chemical) classes**	**Cases *N* = 723**	**Controls *N* = 4,924**	
		***N*** **(%)**	***N*** **(%)**	***p* ^a^**
A	Alimentary tract and metabolism	614 (84.9)	3,994 (81.1)	*0.012*
B	Blood and hematopoietic organs	471 (65.1)	3,085 (62.7)	*0.186*
C	Cardiovascular system	112 (16)	675 (13.7)	*0.193*
D	Dermatological drugs	343 (47.4)	2,131 (43.3)	*0.034*
G	Genitourinary system and sex hormones	253 (35.0)	1,494 (30.3)	*0.011*
H	Systemic hormones	203 (28.1)	1,313 (26.7)	*0.417*
J	General systemic anti-infectives	390 (53.9)	2,421 (49.2)	*0.016*
L	Antineoplastics and immunomodulators	4 (0.6)	9 (0.2)	*0.052*
M	Musculoskeletal system	116 (16)	603 (12.2)	*0.004*
N	**Nervous system (excluding analgesics)**	**87 (12)**	**341 (6.9)**	*** <0.0001***
	Nervous system (including analgesics)	522 (72.2)	3,254 (66.1)	*0.0011*
	**Nervous system (excluding acetaminophen)**	**133 (18)**	**597 (12.1)**	*** <0.0001***
P	Antiparasitic agents, insecticides	47 (7)	300 (6.1)	*0.666*
R	Respiratory system	363 (50.2)	2,350 (47.7)	*0.205*
S	Sensory organs	78 (11)	495 (10.1)	*0.536*

*^a^Significance threshold of statistical tests was 0.0033 (after Bonferroni adjustment)*.

*Bold: ATC classes for which we observed a statistically significant difference of prenatal exposure between cases and controls. Italic: p-values (traditionally written in italic)*.

Table 5 provides a description of the exposure to different subclasses of Nervous System drugs throughout the prenatal period and during each trimester. The proportions of children prenatally exposed to psycholeptics and psychoanaleptics were at least twice as high in the case group as in the control group. The difference between cases and controls prenatal exposure to Nervous System drugs was the highest during the first trimester (*p*<*0.0001*).

**Table 5:** Prenatal exposure of the case and control subjects to ATC (anatomical therapeutic chemical) class *N*.

**ATC (anatomical therapeutic chemical) sub-classes**		**Pregnancy**			**Trimester 1**			**Trimester 2**			**Trimester 3**		
		**Cases *N* = 723**	**Controls *N* = 4,924**		**Cases *N* = 723**	**Controls *N* = 4,924**		**Cases *N* = 723**	**Controls *N* = 4,924**		**Cases *N* = 723**	**Controls *N* = 4,924**	
		***N*** **(%)**		***p* ^a^**	***N*** **(%)**		***p* ^a^**	***N*** **(%)**		***p* ^a^**	***N*** **(%)**		***p* ^a^**
*N* (excluding analgesics)		87 (12.0)	341 (6.9)	* <0.0001*	61 (8.4)	239 (4.9)	* <0.0001*	31 (4.3)	112 (2.3)	*0.0013*	34 (4.7)	116 (2.4)	*0.0002*
N01	Anesthetics	13 (1.8)	78 (1.6)		5 (0.7)	40 (0.8)		2 (0.3)	16 (0.3)		7 (1.0)	31 (0.6)	
N03	Anticonvulsants	6 (0.8)	27 (0.5)		6 (0.8)	22 (0.4)		5 (0.7)	17 (0.3)		5 (0.7)	17 (0.3)	
N05	Psycholeptics	59 (8.2)	201 (4.1)		40 (5.5)	138 (2.8)		18 (2.5)	61 (1.2)		21 (2.9)	53 (1.1)	
N06	Psychoanaleptics	23 (3.2)	70 (1.4)		20 (2.8)	64 (1.3)		13 (1.8)	19 (0.4)		9 (1.2)	15 (0.3)	
N07	Other	4 (0.6)	18 (0.4)		2 (0.3)	16 (0.3)		3 (0.4)	13 (0.3)		1 (0.1)	9 (0.2)	

*^a^Significance threshold of statistical tests was 0.05*.

*Italic: p-values (traditionally written in italic)*.

Table 6 presents the results of univariate and multivariate logistic regressions for the comparison of prenatal nervous system drug exposure between cases and controls. Children in the cases group were significantly more exposed to nervous system drugs during their prenatal life [OR: 2.12; 95% CI (1.55; 2.90)] after adjustment for gender, prematurity, congenital malformation, hearing impairment, maternal age and parity.

**Table 6:** Gross and adjusted odds ratio calculated to compare prenatal nervous system drug exposure between the case and control subjects.

	**Sample size (%)**		**Crude OR^**a**^ (95% CI^**b**^)**	**Adjusted OR (95% CI)**	**Adjusted *P*-value^**d**^**
	**Cases *N* = 723**	**Controls *N* = 4924**			
Prenatal exposure to nervous system drugs	87 (12.0%)	341 (6.9%)	2.07 (1.53; 2.80)	2.12 (1.55; 2.90)^c^	<0.0001

^a^*OR, Odds Ratio*.

*^b^CI, Confidence interval*.

*^c^Adjusted for gender, prematurity, congenital malformation, hearing impairment, maternal age and parity*.

*^d^Significance threshold of statistical tests was 0.05*.

## Discussion

A total of 723 children with a history of neuropsychiatric care were identified, which was 8.6% of the children in the POMME cohort. A comparison of prenatal medication exposure between cases and controls showed a statistically significant difference in nervous system drug exposure, especially when analgesics were excluded.

### Comparison of the Results With Current Knowledge

At a significance threshold of 0.33%, prenatal exposure to other ATC classes than N did not differ statistically in the two groups, but the results point some classes that deserves further investigation. The majority of studies conducted to assess the risk of neurodevelopmental disorders have focused on prenatal exposure to Nervous System drugs, particularly antiepileptics. There is evidence that prenatal exposure to valproate increases the risk of a decrease in Intelligence Quotient (IQ), developmental delay, ASD and ADHD (19–22). Studies on the effect of prenatal exposure to antidepressants or benzodiazepine found conflicting results (6, 23–30). It is therefore still difficult to draw a conclusion on the risk of neuropsychiatric disorders in children after prenatal exposure to all kind of psychotropic medications. One reason for the variability in study results is that the term “neurodevelopmental disorders” encompasses different types of disorders. Moreover, the outcomes considered differ according to the studies: educational support, behavioral disorders, ADHD diagnosis… Our study is innovative in that the criteria for selecting case and control subjects had never been used before because POMME is the only cohort that holds both psychomotor development data for children up to 2 years of age, and data on the reimbursement of medication and care for children during their prenatal life and up to the age of 8 years. Although these criteria do not permit to identify children with specific psychiatric disorders, combining different disorders may be interesting, since it has been shown that numerous neuropsychiatric disorders in children are not isolated but rather associated (31).

The use of the Bonferroni method to consider the increase in the alpha risk is justified because our study was conducted to identify significant associations without any pre-established hypotheses, which is an acceptable condition for use of the Bonferroni correction (32). Our results are reassuring for some ATC classes such as systemic hormones. Nevertheless, the *p*-values calculated when comparing prenatal exposure between case and control subjects were <0.05 for four ATC classes other than N: “M” (Musculoskeletal System), “G” (Genitourinary System and Sex Hormones), “A” (Alimentary Tract and Metabolism), and “J” (Systemic Anti-infectives). For the “M” ATC class, the children in the case group were more exposed to non-steroidal anti-inflammatory drugs (NSAIDs) than those in the control group. Only one study assessed the risk of neuropsychiatric disorders in children prenatally exposed to NSAIDs and the results did not indicate an increased risk (33). To our knowledge, no study has evaluated the risk of neuropsychiatric disorders following prenatal exposure to medications in the “G” and “A” ATC classes. For the “A” class, our study found that the children in the case group were more exposed to antiemetic medications, which have antipsychotic properties that might partly explain the difference observed between case and control subjects. Finally, a few studies found a weak association between prenatal exposure to antibiotics and the occurrence of neuropsychiatric disorders, particularly ASD (34, 35).

### Limits of the Study

The first limitation is inherent to the use of claims databases. The POMME data concern the dispensing and reimbursement of prescribed medications, but not self-medication. Regarding prenatal exposure to Nervous System drugs (excluding analgesics), this may not have led to a classification bias because psychotropics require medical prescription. Similarly, for children on psychotropic medications, the risk of self-medication seems low, considering the age (0–8 years) and the type of medication. Moreover, we cannot know whether the drugs dispensed were effectively consumed by the mother and/or her child. In addition, POMME has missing information on some risk factors (smoking, hearing impairment, alcohol consumption…). Consequently, there may be residual confounding in this study.

The second limitation is that we cannot say that the children in the case group actually have neuropsychiatric disorders. We identified children having a history of neuropsychiatric care. As diagnosis are not available in POMME, we carefully elaborated inclusion and exclusion criteria to identify children with a high probability of having neuropsychiatric disorders. This work was done in collaboration with child psychiatrists. Children identified as cases received care or treatment for neurodevelopmental or psychiatric disorders, which may be more sensitive and may allow to identify children at an earlier stage than the diagnosis. Indeed, a recent study showed that children with neurodevelopmental disorders and disabilities have higher healthcare service utilization than those without (36). We used a body of criteria to identify children for whom data from health certificates were missing, or who did not consult any neuropsychiatric specialist, or to whom no psychotropic medication were prescribed. Revet et al. conducted a systematic review to describe the use of the French national health insurance information system for research in the field of mental health (37). They found that only two studies aimed to validate specific psychiatric disorders' identification algorithms (for schizophrenia and depression), but no study focused on the development of algorithms to identify pediatric psychiatric disorders. Consequently, no algorithm was available to identify cases in our study. Moreover, we used the psychomotor development items as criteria, which is innovative because no database in France records these data. The next step will be to perform a study aiming at validate our case selection algorithm.

The third limitation is that neuropsychiatric disorders encompass heterogeneous conditions, including neurodevelopmental and psychiatric disorders. Although numerous studies have already been conducted to evaluate the association between prenatal exposure to psychotropic medications and specific neuropsychiatric disorders (e.g., ADHD or ASD…), our approach using a composite outcome addresses knowledge gap in the literature regarding other medication classes than psychotropics. Further studies are needed to assess the risk of specific neuropsychiatric disorders and prenatal exposure to medications belonging to the ATC classes: Musculoskeletal System, Genitourinary System and Sex Hormones, Alimentary Tract and Metabolism, and Systemic Anti-infectives.

The main bias identified in this study was an indication bias. In fact, the type of pathology for which the mother took a psychotropic medication as well as the severity of the pathology probably has an influence on the child's neuropsychiatric development (23). Maternal psychiatric disorders could not be specifically identified in POMME but our model was adjusted on the variable “long-term disease”. In France, for the patients diagnosed with any chronic condition specifically listed by the French healthcare system as a long-term disease (18), medical care related to the pathology are provided free of charge. The list includes psychiatric disorders.

Prenatal medication exposure was higher in the case group than in the control group for all the ATC classes. One of the reasons could be the propensity of mothers who take a lot of medications to ask for more medications (and more medical care) for their children. Certain other factors could not be considered. For example, socio-economic level and maternal IQ were not available, while studies have shown that childhood neurodevelopment is related to these different factors (38, 39). The maternal level of education and the parents' profession could be taken into account in our analyses. Although there were missing data for these covariates, this limits confusion bias.

### Strengths of the Study

This is the first study using child psychomotor development data from health certificates to identify cases and controls. The recommendations made in 2018 by the French Health Agency to improve the diagnosis of ASD specified that the items of psychomotor development should not be analyzed in isolation, but through a combination of at least two signs, which requires clinical screening of the child's development (40). For this reason, criteria for case selection included the presence of at least two negative psychomotor development items at 9 months and/or two negative items at 24 months. The analysis of the health certificates of the cases revealed that their psychomotor development was disrupted as early as 9 months of age. Therefore, psychomotor development items are early warning signs of a psychomotor development disorder. Medical visits to neuropsychiatric specialists were also among our criteria for selecting cases. This criterion enables early identification of children who show signs of abnormal psychomotor development and for whom a diagnosis has not yet been made. Similarly, if a neuropsychiatric condition is specified on the open fields of the medical certificate, it can be a sign of either a suspicion from the physician or a diagnosis. Finally, we identified cases through prescriptions of medications indicated for neuropsychiatric disorders. This criterion is essential but not sufficient because the use of psychotropic medications in children is low, especially in France (41).

The POMME cohort appears to be a representative sample of French live births because children characteristics are similar to those of the children included in the French national perinatal survey, that was published in 2010 (see Supplementary Table 2) (42).

Finally, this exploratory study is one of the first to examine prenatal exposure to all ATC classes, without any initial hypothesis. The association between prenatal exposure to psychotropic medications and childhood neuropsychiatric disorders is biologically plausible because psychotropic medications act on the central nervous system and cross the blood-brain barrier. Since properties of drugs that enable them to cross the blood-brain barrier (small size, lipophilicity) are similar to those required to cross the placental barrier, these medications easily reach the fetal compartment. Their effects on the nervous system of the developing and maturing fetus can have long-term consequences on the functioning of the brain (43). Yet, many medications cross the placenta and it is not because their principal mechanism does not involve the central nervous system that they may not lead to delayed neuropsychiatric adverse effects.

In conclusion, this study shows that children with neuropsychiatric disorders were prenatally more exposed to the ATC class of Nervous System drugs than controls and provides clues for conducting additional studies to evaluate the risk of neuropsychiatric disorders in children subsequent to prenatal exposure to medications.

## Data Availability Statement

The raw data supporting the conclusions of this article will be made available by the authors upon reasonable request and with permission of the Haute-Garonne Health Insurance Service and the Haute-Garonne Maternal and Child Protection Service.

## Ethics Statement

The studies involving human participants were reviewed and approved by CNIL (Commission Nationale de l'Informatique et des Libertés), DR-2013-060. This study was performed on anonymized patient data. The mothers of the child included in POMME were informed of their inclusion and of the potential use of their anonymized data for research purposes. They can oppose the use of their data at any time.

## Author Contributions

JB had full access to all the data in the study and takes responsibility for the integrity of the data and the accuracy of the data analysis. JB, CD-M, IL, AR, and AS: concept and design. JB, CD-M, and CH-D: data collection, analysis, and interpretation of data. JB and CD-M: drafting the manuscript. JB, CH-D, and MA: statistical analysis. CD-M: supervision. All authors made substantial contributions to the conception or design of the work, or the acquisition, analysis, or interpretation of data, or the creation of new software used in the work, drafted the work or revised it critically for important intellectual content, approved the version to be published, agree to be accountable for all aspects of the work in ensuring that questions related to the accuracy or integrity of any part of the work are appropriately investigated and resolved, and critical revision of the manuscript for important intellectual content.

## Conflict of Interest

The authors declare that the research was conducted in the absence of any commercial or financial relationships that could be construed as a potential conflict of interest.

## Publisher's Note

All claims expressed in this article are solely those of the authors and do not necessarily represent those of their affiliated organizations, or those of the publisher, the editors and the reviewers. Any product that may be evaluated in this article, or claim that may be made by its manufacturer, is not guaranteed or endorsed by the publisher.
