# Supplementary material for: Prenatal Drug Exposure in Children With a History of Neuropsychiatric Care: A Nested Case-Control Study
Source: Front Psychiatry. 2022 Mar 22;13:795890. doi: 10.3389/fpsyt.2022.795890 (PMC8980541; doi:10.3389/fpsyt.2022.795890)
Supplement: Supplementary file 1 [file Data_Sheet_1.docx]

## Manuscript Title: Prenatal Drug Exposure in Children with a History of Neuropsychiatric Care: A Nested Case-Control Study

Journal: Frontiers in Psychiatry

**Author information**: Justine Benevent^a^, Caroline Hurault-Delarue^a^, Mélanie Araujo^a^, Alexis Revet ^a,b^, Agnès Sommet ^a^, Isabelle Lacroix ^a^, Christine Damase-Michel ^a^

**Authors’ affiliations:**

^a^ Department of Medical and Clinical Pharmacology, Toulouse Faculty of Medicine, CERPOP - SPHERE team, Toulouse University, Inserm, Université Paul Sabatier, Toulouse, France

^b^ Department of Child and Adolescent Psychiatry, Toulouse University Hospital (CHU de Toulouse), Toulouse, France

**Corresponding author**: Justine Benevent, Email: [justine.benevent@univ-tlse3.fr](mailto:justine.benevent@univ-tlse3.fr)

Online only table

Supplementary Table 1: Congenital malformations observed in the case and control subjects

| **Congenital malformation (major or minor)** | **CASES (N=723)** | | **CONTROLS (N=4,924)** | |
| --- | --- | --- | --- | --- |
|  | **N** | **Rate of malformation (per 10,000)** | **N** | **Rate of malformation (per 10,000)** |
| **All abnormalities** | **37** | **510.3** | **89** | **180.7** |
| **Nervous system** | **3** | **41.4** | **2** | **4.1** |
| Microcephaly | 1 | 13.8 | 0 | 0 |
| Congenital hydrocephalus | 1 | 13.8 | 1 | 2.0 |
| Other congenital malformations of brain | 1 | 13.8 | 0 | 0 |
| Other congenital malformations of nervous system | 0 | 0 | 1 | 2.0 |
| **Eye, ear, face and neck** | **1** | **13.8** | **5** | **10.2** |
| Congenital malformations of eyelid, lacrimal apparatus and orbit | 1 | 13.8 | 0 | 0 |
| Congenital lens malformations | 0 | 0 | 3 | 6.1 |
| Congenital malformation of ear causing impairment of hearing | 0 | 0 | 1 | 2.0 |
| Other congenital malformations of ear | 0 | 0 | 2 | 4,1 |
| **Circulatory system** | **7** | **96.6** | **7** | **14.2** |
| Malformations of cardiac septa | 0 | 0 | 1 | 2.0 |
| Congenital malformations of pulmonary and tricuspid valves | 0 | 0 | 1 | 2.0 |
| Other congenital malformations of heart | 7 | 96.6 | 4 | 8.1 |
| Congenital malformations of great arteries | 0 | 0 | 1 | 2.0 |
| Congenital malformations of great veins | 0 | 0 | 1 | 2.0 |
| **Respiratory system** | **2** | **27.6** | **3** | **6.1** |
| Congenital malformations of nose | 0 | 0 | 1 | 2.0 |
| Congenital malformations of larynx | 1 | 13.8 | 2 | 4.1 |
| Congenital malformations of lung | 1 | 13.8 | 0 | 0 |
| **Cleft lip and cleft palate** | **3** | **41.4** | **1** | **2.0** |
| Cleft palate | 1 | 13.8 | 0 | 0 |
| Cleft lip | 0 | 0 | 1 | 2.0 |
| Cleft palate with cleft lip | 2 | 27.6 | 0 | 0 |
| **Alimentary tract** | **0** | **0** | **6** | **12.2** |
| Other congenital malformations of tongue, mouth and pharynx | 0 | 0 | 1 | 2.0 |
| Congenital malformations of esophagus | 0 | 0 | 1 | 2.0 |
| Other congenital malformations of upper alimentary tract | 0 | 0 | 1 | 2.0 |
| Other congenital malformations of intestine | 0 | 0 | 3 | 6.1 |
| **Genitalia** | **5** | **69** | **20** | **40.6** |
| Congenital malformations of ovaries, fallopian tubes and broad ligaments | 0 | 0 | 2 | 4.1 |
| Other congenital malformations of female genitalia | 0 | 0 | 1 | 2.0 |
| Undescended testicle | 3 | 41.4 | 5 | 10.2 |
| Hypospadias | 0 | 0 | 9 | 18.3 |
| Other congenital malformations of male genitalia | 2 | 27.6 | 4 | 8.1 |
| **Urinary system** | **12** | **165.5** | **14** | **28.4** |
| Renal agenesis and other reduction defects of kidney | 1 | 13.8 | 3 | 6.1 |
| Cystic kidney diseases | 3 | 41.4 | 0 | 0 |
| Congenital obstructive defects of renal pelvis and congenital malformations of ureter | 4 | 55.2 | 3 | 6.1 |
| Other congenital malformations of kidney | 1 | 13.8 | 2 | 4.1 |
| Other congenital malformations of urinary system | 3 | 41.4 | 6 | 12.2 |
| **Musculoskeletal system** | **4** | **55.2** | **27** | **54.8** |
| Congenital deformities of hip | 0 | 0 | 6 | 12.2 |
| Congenital deformities of feet | 1 | 13.8 | 7 | 14.2 |
| Congenital musculoskeletal deformities of head, face, spine and chest | 2 | 27.6 | 2 | 4.1 |
| Other congenital musculoskeletal deformities | 0 | 0 | 3 | 6.1 |
| Polydactyly | 0 | 0 | 1 | 2.0 |
| Syndactyly | 0 | 0 | 1 | 2.0 |
| Reduction defects of upper limb | 0 | 0 | 1 | 2.0 |
| Other congenital deformities of skull and face bones | 1 | 13.8 | 2 | 4.1 |
| Congenital malformations of spine and bony thorax | 0 | 0 | 3 | 6.1 |
| Congenital malformations of the musculoskeletal system, not elsewhere classified | 0 | 0 | 1 | 2.0 |
| **Other congenital malformations** | **1** | **13.8** | **7** | **14.2** |
| Other congenital malformations of skin | 0 | 0 | 5 | 10.2 |
| Congenital malformation syndromes due to known exogenous causes, not elsewhere classified | 1 | 13.8 | 1 | 2.0 |
| **Other congenital malformations, not elsewhere classified** | **2** | **27.6** | **1** | **2.0** |
| Other chromosome abnormalities, not elsewhere classified | 0 | 0 | 1 | 2.0 |
| Down syndrome | 1 | 13.8 | 1 | 2.0 |
| Other sex chromosome abnormalities, male phenotype, not elsewhere classified | 1 | 13.8 | 0 | 0 |

Supplementary Table 2: Comparison of indicators between the 2010 national perinatal survey and the POMME cohort

|  | **POMME-2010**  **N=8,372** | **French national perinatal survey - 2010** |
| --- | --- | --- |
| *Characteristics* | *n(%)* | *%* |
| Male^†^ | 4,248(50.7%) | 52.3 |
| Preterm birth (gestational age <37 weeks) ^†^ | 519(6.2%) | 6.5 |
| Breastfed infant | 5,552(66.3%) | 68.7 |
| Intubation^†^ | 51(0.6%) | 1.1 |
| Mother has no profession | 1,948(23.3%) | 26.7 |
| Maternal level of education (N=4094) |  |  |
| Primary school | 62(1.5%) | 2.4 |
| High school | 540(13.3%) | 25.7 |
| High school diploma level | 898(21.9%) | 19.8 |
| Higher education | 2,627(64.2%) | 52.1 |
| *Characteristics* | *Mean±SD* | *Mean±SD* |
| Maternal age | 30.6±5.0 | 29.7±5.4 |
| Head circumference at birth (cm) ^†^ | 34.3±1.6 | 34.3±1.9 |
| Birth weight (g) ^†^ | 3,260.9±509.9 | 3,272.3±529.5 |
| Birth height (cm) ^†^ | 49.1±2.4 | 49.3±2.5 |

^†^These characteristics are calculated based on different populations since the national perinatal survey includes all births (live births, stillbirths, fetal deaths) and the POMME cohort includes only livebirths.
